# Viral myocarditis vs lupus myocarditis, distinctive features in Cardiovascular Magnetic Resonance

**DOI:** 10.1186/1532-429X-18-S1-O97

**Published:** 2016-01-27

**Authors:** Maria A Espinoza Barillas

**Affiliations:** Radiology, Instituto Nacional De Ciencias Medicas Y Nutricion Salvador Zubiran, Mexico DF, Nicaragua

## Background

Myocardial inflammation may have multiple etiologies; infectious, toxic, and autoimmune. Acute cases are mostly due to myocardial involucre of a systemic viral infection. Although infectious myocarditis is routine in clinical practice for cardiologists, myocarditis presented by autoimmune diseases such as systemic lupus erythematosus also should be considered.

## Methods

RMC was performed in 59 patients with suspected myocarditis: 30 patients with diagnosis of SLE based on the criteria of the American College of Rheumatology and symptoms and signs of lupus activity evidenced by ECLAM (European Consensus Lupus Activity Measurement) scale with or without cardiovascular symptoms, we excluded patients with SLE and recent intake of drugs or other substances associated with myocarditis,renal function failed and history of ischemic heart disease; And 29 patients that were assessed by certified cardiologists who suspected viral myocarditis due to the combination of the following factors: symptoms (chest pain, dyspnea and malaise), previously healthy and with no history of cardiovascular disease, with or without history of infection (upper respiratory tract infection and / or gastroenteritis) in the last 4 weeks. patients with coronary angiotomography and / or angiography for positive for atherosclerotic significant disease and patients with positive myocardial ischemia test.

## Results

The most common clinical presentation in patients with lupus was dyspnea (83%), in patients with viral myocarditis the symptom was chest pain (72%) of them 62% had a history of a recent viral illness. In lupus myocarditis there was greater involvement of the ejection fraction of the left ventricle, pericardial (pericardial effusion 60% and 73% of patients with pericarditis) and valvulitis (86%) involvement, characteristics that were not identified in viral myocarditis. Lake Louis criteria; in both groups relative and global enhancement were most often negative,relative enhancement positivity percentage was 44 and 33 respectively for viral myocarditis and lupus. The global enhancement was positive in only 31% of lupus cases and 44% of viral. The gadolinium contrast enhancement was positive in the majority of lupus patients and 63% and in less than half of patients with viral myocarditis (41%). The location of these lesions had a similar behavior in both etiologies, anterior and inferoseptal being the most affected segments.

## Conclusions

Lupus myocarditis unlike viral myocarditis, characteristically presents pancarditis with pericardial and valvular involvement, and most often affected the ventricular function. The late enhancement was the criteria that prevailed in lupus myocarditis. There were no differences in the location of delayed enhancement lesions between the two etiologies.

**Figure 1:**
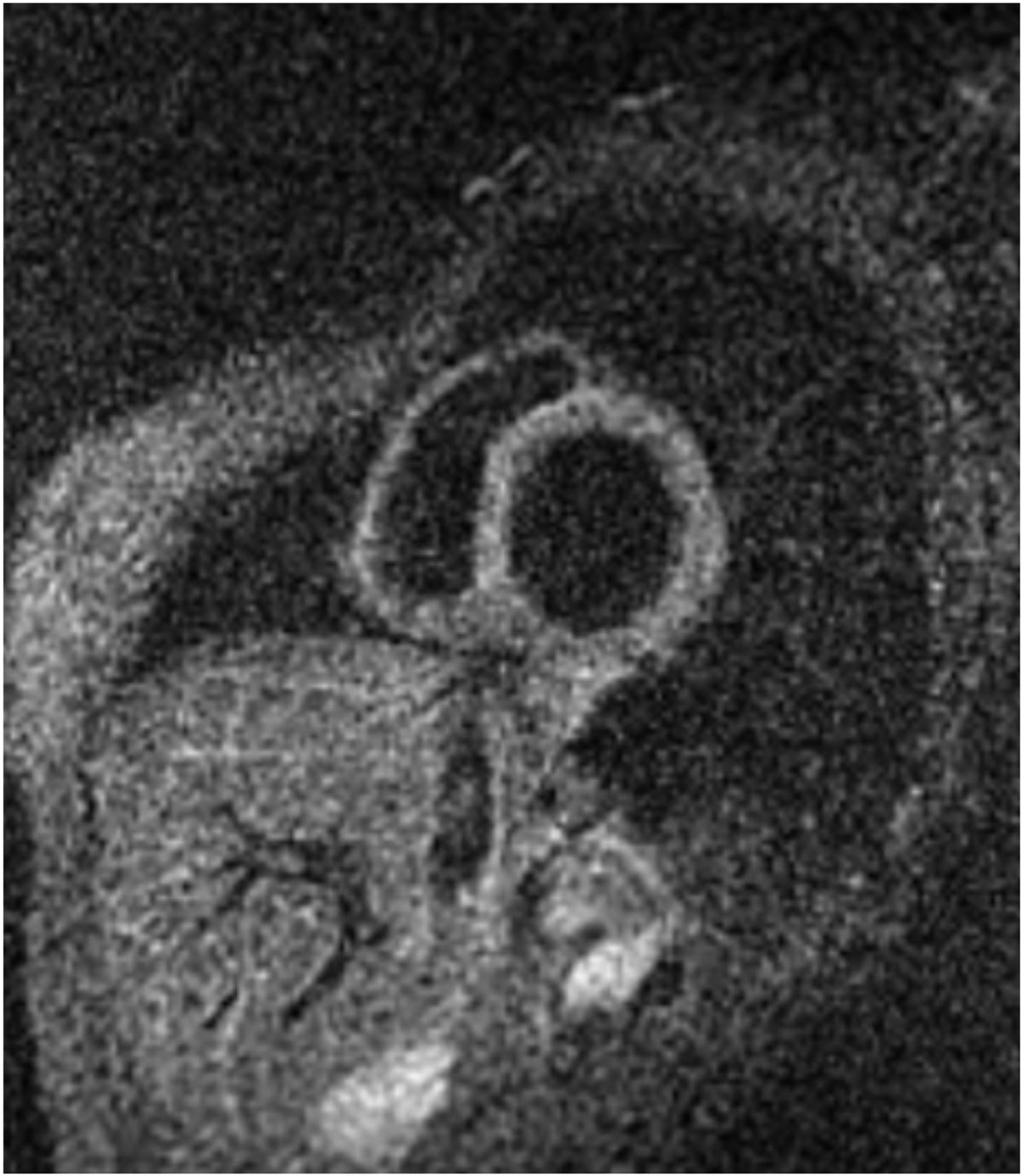
**SLE patient**. T2 weighted (STIR) in short axis positive for myocardial edema.

